# Digital tools for youth mental health

**DOI:** 10.1038/s41746-019-0181-2

**Published:** 2019-10-18

**Authors:** Peter Uhlhaas, John Torous

**Affiliations:** 1 Institute of Neuroscience and Psychology, University of Glasgow, 58 Hillhead Street, Glasgow, G12 8QB Scotland UK; 2 Charité Universitätsmedizin, Department of Child and Adolescent Psychiatry, Berlin, Germany; 3 Division of Digital Psychiatry, Beth Israel Deaconess Medical Center, Harvard Medical School, Boston, MA 02446 USA

**Keywords:** Rehabilitation, Diagnostic markers

Mental disorders, such as schizophrenia, depression, anxiety, personality disorders, and substance abuse, remain a leading source of disability, healthcare expenditure, and personal suffering in all countries. It is estimated that 1.1 billion people are affected by mental or substance use disorders worldwide and significant treatment gaps in both high and middle-income countries.^1^

A critical aspect of mental disorders is that the majority of mental ill-health occurs during youth, here defined as the period between 15 and 24 years. Thus, emerging evidence suggests that the large majority (75%) of mental disorders have their onset between 15 and 24 years of age.^2^ Importantly, this is also accompanied by high degree of access to digital devices in this age group which could provide a unique opportunity to develop scalable and accessible digital approaches for mental health. For example, in this same age group, there is 90% smartphone prevalence of ownership in high-income countries and over 50% in low- and middle-income countries with the majority around the world using social media (https://www.pewresearch.org/global/2019/02/05/in-emerging-economies-smartphone-adoption-has-grown-more-quickly-among-younger-generations/).

From a biological and clinical perspective, it is has been proposed that early detection and intervention for mental health in youth may hold great promise for improving long-term outcomes.^3^ One reason for the absence of significant breakthroughs in improving mental health has been the way in which research and treatments have been framed. Over the last 100 years, a cardinal feature of the existing paradigm in mental health has been its emphasis on fully established disorders in adulthood. However, a significant paradigm shift has emerged over the last decade or so aimed at improving mental health, in particular in young people/youth, stimulated by the early intervention approach in the psychosis movement.^4^

This paradigm shift in mental health has significant implications in the way mental disorders are identified, as well as treatment are delivered in this age. First, there is a growing recognition that a profound redesign of services and intervention is required that reflect the specific needs of service users. Second, novel approaches are required to facilitate the identification of emerging mental disorders at the sub-syndromal level and risk factors for later development of fully fledged syndromes that are a crucial pre-requisite for the early intervention approach. Thus, we would like to make the case that digital medicine and its resulting approach and tools may be crucially relevant for youth mental health in regards to two key aspects: (1) detection of emerging mental disorders; and (2) providing youth-friendly treatment approaches that leverage digital approaches, as well as technologies.

Today’s challenges around youth mental health provide an ideal opportunity for digital augmentation. Efficient methods for the detection and early diagnosis are critical—but current health systems provide significant barriers towards the implementation of early intervention. This is because current models of care and diagnosis create significant obstacles for detecting precursors for mental ill-health, in particular during youth, as the result of stigma and continued underfunding of services.^5^ Rather than force healthcare system to embrace public health roles, they are often not equipped to undertake—digital approaches could complement current mental health systems. For example, digital spaces for help-seeking youth could provide the first entry points for clinical services and education. In a recent study, we developed a web-based screening platform for emerging signs of psychosis that employed a population-wide online-screening approach.^6^ The results showed that the website was highly endorsed by participants and allowed the identification of both youth with at-risk symptoms as well as signs of fully manifested psychosis with good sensitivity and specificity. Accordingly, these findings highlight the potential of detecting emerging as well as manifest mental ill-health in youth through digital approaches that otherwise may not be detected by conventional services. We feel that the scalability of such screening approaches could constitute a crucial approach for the detection of emerging psychosis but also other aspects of mental health outside traditional care-pathways, such as capturing the personal environmental, social, and physiological predictors of suicidal ideations to enable more precise and accurate risk assessment.^7^ A critical aspect of this approach, however, is that these digital platforms are linked with front-line clinical services, as well as are characterized by sufficient sensitivity and specificity for predicting clinical status and outcomes. These objectives remain to be established and will provide significant challenges for digital approaches in youth mental health.

In addition to connecting youth who are characterized by emerging and/or fully symptoms of mental ill-health with care, digital health and tools can also help monitor recovery, provide early warnings signs of risk or relapse, and offer novel information on functional outcomes. For example, with digital phenotyping—the moment-by-moment quantification of the individual-level human phenotype in situ using data from personal digital devices—it is now possible to capture not only that a young person had hallucinatory symptoms but also the environment, social context, and even autonomic response surrounding the experience.^8^ In one study, we were able to predict relapse in patients with psychosis through applying anomaly detection to captured personal changes in mobility, socialness, and self-reported symptoms up to 2 weeks before clinical relapse.^9^ Having a stream of multimodal, dynamic, longitudinal, and real-time digital biomarkers will help advance not only understanding the emergence of symptoms during youth but may also provide novel therapeutic opportunities.^10^

Related to monitoring, a second crucial area for digital medicine as applied to youth mental health is the delivery of interventions. Despite increased attention towards global mental health, the large majority of people experiencing mental health problems continue not to receive adequate care. For example, only one in five people from high-income countries and only one in 27 people from low- and middle-income countries receive minimally adequate treatment for depression.^11^ This is particularly the case for youth who despite the high prevalence of mental disorders face major difficulties in accessing appropriate treatment.^12^ This is partly due to the “gap” in care between child and adult mental health services as well as to a reluctance to seek help, stigma, and negative attitudes towards professionals.^13,14^ As a result, less than one in five adolescents with disorders of youth onset (anxiety, substance use disorders) received care.^15^

Digital medicine offers several potential avenues of delivering services and augmenting interventions, including text messaging, web, smartphone app, and virtual reality. Each technology presents a unique potential for youth mental health, although none is a panacea. Text messaging is easily scalable and can work on nearly any mobile phones today—with youth willing to engaging around mental health text messages.^16^ There is a rich legacy of web-based programs for youth mental health^17^ and a stream of new tools using today’s social networking features.^18^ Computer and video games are also receiving recognition as tools to improve mental health^19^ and hold unique potential given their high rates of engagement. One game is even likely to receive FDA approval for ADHD,^20^ suggesting therapeutic video games for youth mental health are likely to rapidly expand. App-based technology interventions are nascent, but rapidly evolving. For example, a 2019 review of smartphone apps targeting child and adolescent psychiatry found only six studies that met strict inclusion criteria.^21^ Reviews of apps for youth mental health currently available for download on the commercial marketplaces have also raised concerns about lack of quality and adherence to evidence-based practices.^22^ But while today’s apps tools for youth mental health may have room to improve, a new wave of research and tools in development harbingers a second generation of impactful apps.

But regardless of technology modality employed, challenges around privacy and ethics cannot be ignored. As we are able to capture more real-time data on youth mental health, and reach out with real-time/just in time interventions, how do we ensure care remains safe and youth are able to make informed decisions about use? For example, today Facebook conducts automatic suicide screenings and sends police to peoples’ homes, despite no evidence of the effectiveness or data on risks, and with no easy way for youth to understand, opt in/out, or consent to this use of their personal data.^23^ While it is critical that innovation in youth mental health not be hampered, rushing too fast and violating the basic rights of young people is a sure way to ensure digital medicine will never reach its potential and that youth will refuse to use it. Creating digital ecosystems of innovation and transparency represents the new paradigm shift the field must embrace. The need for co-designing this new generation of digital mental health tools is finally becoming well recognized and user-cantered design is quickly becoming the new normal. While the debate on the impact of screen time on the developing brain and [youth] mental health remains ongoing and requires further investigation^24^—it would be foolish and irresponsible to ignore the potential of the digital world itself on youth mental health. Implementation challenges and the merging of digital medicine and mental health present friction, but with youth mental health those barriers may be lighter as younger patients come to expect at least some aspects of technology in their care.

Like a ball rolling downhill with gravity determining exactly where it will rest, so the future of youth mental health will rest with digital medicine. The current unmet need combined with prevalence and potential of digital technologies is the guiding force, although the actual pathway towards that future is less clear. A smooth transition with new information about the developing brain and youth mental health could inform a new generation of digital interventions, which is used ethically could advance the field in mere years. But the transition could be bumpier with false starts and ethical challenges. The articles in this special edition help guide the field towards the smoother transition path and guide the way with novel investigations and insightful perspectives.
